# Cranial CT is a mandatory tool to exclude asymptomatic cerebral hemorrhage in elderly patients on anticoagulation

**DOI:** 10.3389/fmed.2023.1117777

**Published:** 2023-01-27

**Authors:** Cora R. Schindler, Alicia Best, Mathias Woschek, René D. Verboket, Ingo Marzi, Katrin Eichler, Philipp Störmann

**Affiliations:** ^1^Department of Trauma, Hand and Reconstructive Surgery, University Hospital Frankfurt, Frankfurt am Main, Germany; ^2^Department of Diagnostic and Interventional Radiology, University Hospital Frankfurt, Frankfurt am Main, Germany

**Keywords:** TBI, anticoagulation, cerebral hemorrhage, elderly, geriatric TBI, computertomografie

## Abstract

**Background:**

Traumatic brain injury (TBI) after falls causes death and disability with immense socioeconomic impact through medical and rehabilitation costs in geriatric patients. Diagnosing TBI can be challenging due to the absence of initial clinical symptoms. Misdiagnosis is particularly dangerous in patients on permanent anticoagulation because minimal trauma might result in severe intracranial hemorrhage. The aim of this study is to evaluate the diagnostic necessity of cranial computed tomography (cCT) to rule out intracranial hemorrhage, particularly in the absence of neurologic symptoms in elderly patients on permanent anticoagulation in their premedication.

**Patients and methods:**

Retrospective cohort analysis of elderly trauma patients (≥ 65 years) admitted to the emergency department (ED) of the level-1-trauma center of the University Hospital Frankfurt from 01/2017 to 12/2019. The study included patients who suffered a ground-level fall with suspected TBI and subsequently underwent CT because of preexisting anticoagulation.

**Results:**

A total of 227 patients met the inclusion criteria. In 17 of these patients, cCT showed intracranial hemorrhage, of which 14 were subdural hematomas (SDH). In 8 of the patients with bleeding showed no clinical symptoms, representing 5% (*n* = 160) of all symptom-free patients. Men and women were equally to suffer a post-traumatic hemorrhage. Patients with intracranial bleeding were hospitalized for 14.5 (±10.4) days. Acetylsalicylic acid (ASA) was the most prescribed anticoagulant in both patient cohorts—with or without intracerebral bleeding (70.6 vs. 77.1%, *p* = 0.539). Similarly, patients taking new oral anticoagulant (NOAC) (*p* = 0.748), coumarins, or other platelet inhibitors (*p* > 0.1) did not show an increased bleeding incidence.

**Conclusion:**

Acetylsalicylic acid and NOAC use are not associated with increased bleeding risk in geriatric trauma patients (≥ 65 years) after fall-related TBI. Even in asymptomatic elderly patients on anticoagulation, intracranial hemorrhage occurs in a relevant proportion after minor trauma to the head. Therefore, cCT is an obligatory tool to rule out cerebral hemorrhage in elderly patients under anticoagulation.

## Introduction

Severe traumatic brain injury (TBI) caused by road traffic accidents and falls are the overall main causes of death and disability with immense socioeconomic impact through loss of productivity as well as medical and rehabilitation costs (1–3). About one-third of seniors older than 65 years of age fall at least one time a year, and 60–70% of them fall again within a year. Along with fall-related fractures, TBI is one of the most common injury patterns caused by minor injury mechanisms like ground level falls in this population (4, 5).

In TBI, primary brain damage occurs due to rupture of vessels and direct damage to brain tissue which results subsequently in cerebral hemorrhage, axonal shear injury and secondary brain damage, like cerebral edema. In these cases, a phasic course can be observed clinically, with initial loss of conciseness (LOC), transient clearing, and secondary unconsciousness (6). In addition to irreversible primary brain damage by cell death, the outcome after TBI is largely determined by secondary brain damage due to hypoxia or intracranial pressure (7, 8). Therefore, prompt diagnosis and appropriate treatment are crucial to achieve optimal outcome.

Major clinical symptoms of brain injury include, i.e., LOC, amnesia, decreased vigilance or vomiting. Subjective minor symptoms are headache, nausea, dizziness, or double vision. Primary mild or even absent symptoms can complicate the diagnosis of relevant TBI because injury severity does not always correlate with the extent of the initial functional impairment (6, 9).

The diagnostic and therapeutic approach to TBI is initially based on the accident mechanism, the presence and severity of neurologic symptoms, and furthermore depends on existing risk factors. Patients without neurological symptoms and corresponding risk factors can be monitored clinically without radiological diagnostics (10). In the presence of neurological symptoms and/or risk factors, native cranial computed tomography (cCT) is considered the gold standard in the primary diagnosis of TBI with respect to the assessment of intracranial damage. In addition to its high sensitivity and specificity, cCT has short examination times and is ubiquitously available (11, 12). A disadvantage of cCT is the radiation exposure to the patient with an average effective dose of 2.6 mSv. Since delayed or undiagnosed intracranial injuries lead to high subsequent costs due to permanent health damage, cCT is also cost-effective when correctly indicated (13).

Older patients are often treated for vascular or cardiac disease with regular use of anticoagulant medications to prevent and/or treat thromboembolic events. These drugs include antiplatelet agents [e.g., acetylsalicylic acid (ASA)], new oral anticoagulants (NOACs), coumarins (vitamin K antagonists), unfractionated and low-molecular-weight heparins (UFH, LMWH), Especially in Anglo-American countries, but also in Germany, the use of ASA for primary prevention of cardiovascular diseases is widespread. Among them, almost half of those over 70 years of age take ASA daily (14). All substance groups inhibit physiological blood clotting in different ways and thus generally increase the risk of bleeding following trauma. It is particularly dangerous to underestimate the severity of a TBI in patients on anticoagulant medication due to probable relevant progression of an intracranial hematoma caused by the insufficient blood clotting (10, 12). While there is a growing consensus and S1-guidelines for the diagnosis and care of patients with TBI, management in older patients, particularly those taking anticoagulant medications, remains elusive due to a lack of evidence (9).

Therefore, the aim of this study is to evaluate the diagnostic value of cCT regarding intracranial hemorrhage particularly in the absence of neurologic symptoms in elderly patients on common permanent anticoagulation.

## Materials and methods

### Patients and study setting

We retrospectively reviewed the cohort of geriatric trauma patients (≥ 65 years) admitted to the level-1-trauma center of the University Hospital Frankfurt from 01/2017 to 12/2019. The following inclusion criteria were defined: All patients aged ≥ 65 years on long-term anticoagulant medication admitted to the emergency department (ED) after minor trauma (ground-level fall) with suspected TBI. In addition, all included patients underwent cCT because of their anticoagulant medication, regardless of whether they had symptoms of TBI. Patients with suspected severe injuries due to the trauma mechanism who were referred to trauma bay were excluded from the analysis.

The analysis is based on a detailed retrospective review of patient charts evaluating demographic and clinical data. This further includes information on injury patterns, comorbidities, prehospital and in-hospital management, and the process of care in the hospital, as well as examination, laboratory results and outcome data.

### Ethics

The study was performed at the University Hospital Frankfurt, Goethe University after approval by the Institutional Review Board (2021-90) in accordance with the Declaration of Helsinki and following STROBE guidelines and the RECORD guidelines for observational studies (Reporting of studies Conducted using Observational Routinely Collected Data) (14, 15).

### Statistical analysis

Continuous normally distributed variables were summarized using means ± standard deviation (SD). Values are reported as mean for continuous variables and as percentages for categorical variables. The *p*-values for categorical variables were derived from the two-sided Fisher’s exact test, and for continuous variables from the Mann–Whitney U test. Significant values were adjusted by the Bonferroni *post hoc* test. A *p*-value < 0.05 was considered to be statistically significant (*p < 0.05; ^**^p < 0.01; ^***^p < 0.001). All analyses were performed using the Statistical Package for Social Sciences (SPSS for Mac©), version 26 (SPSS Inc., Chicago, IL, USA).

## Results

During the 36-month study period, *n* = 227 patients met the inclusion criteria (≥ 65 years, multimorbidity, TBI, cCT, minor trauma mechanism and anticoagulant therapy). In *n* = 17 (7.5%) patients, cranial CT scan on the day of admission to the ED revealed post-traumatic intracranial hemorrhage.

### The incidence of post-traumatic hemorrhage in the elderly is not related to gender

Table 1 shows demographic and clinical characteristics stratified by the incidence of post traumatic intracranial bleeding. Men and women were almost equally likely to suffer a post-traumatic hemorrhage, and the mean of age of both cohorts was about 81 years. All patients with proven bleeding were hospitalized with a mean length of stay (±SD) of 14.5 (±10.4) days. In total, 17.6% of these patients spent 2.2 (±7.0) days on Intensive Care Unit (ICU).

**TABLE 1:** Demographic and clinical characteristics stratified by the incidence of post-traumatic intracranial bleeding.

	Bleeding		
	**Positive** ***n* = 17**	**Negative** ***n* = 210**	***p*-value**
Sex (male; %)	41.2	47.1	0.64
Age (years; mean ± SD)	81 ± 10	81 ± 7	0.67
Outpatient (%)	0	69.0	<0.001
Inpatient (%)	100	31.0	<0.001
Hospitalization (days; mean ± SD)	14.5 ± 10.4	2.2 ± 4.6	<0.001
ICU (%)	17.6	0	<0.001
ICU (days, mean ± SD)	2.2. ± 7.0	0	<0.001
Mortality (%)	5.9	0	<0.001
**Co-morbidity**			
Cardiac (%)	82.4	81.9	0.22
Neurologic (%)	0	9.5	0.38
**Coagulation parameters**			
Quick (%; mean ± SD)	91.7 ± 22.5	87.6 ± 1.2	0.31
INR (mean ± SD)	1.1 ± 0.4	1.2 ± 0.42	0.36
PTT (s; mean ± SD)	28.94 ± 3.2	27.9 ± 5.1	0.15
Thrombocytes (/μl; mean ± SD)	257 ± 92	223 ± 69	0.1

In comparison, patients without hemorrhage were less often hospitalized (31% of *n* = 210, *p* < 0.001) and had a significantly shorter in-hospital stay of 2.2 (±4.6) days (*p* < 0.001). None of them were monitored on ICU (*p* < 0.001).

In both groups, relevant previous diseases were documented in the medical history, which were mainly of cardiac entity (> 80%). Mean laboratory coagulation parameters (Quick, INR, PTT) showed normal values in both groups.

### ASA and NOAC use are not associated with increased bleeding risk in geriatric trauma patients

**FIGURE 1:**
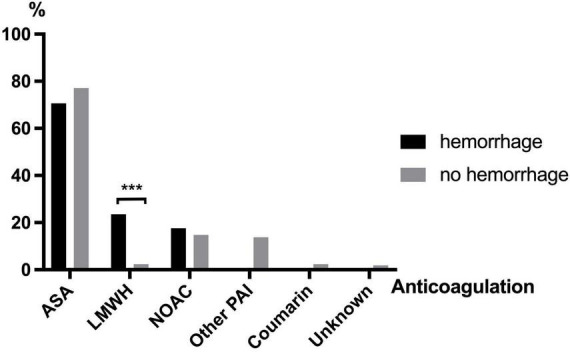
Anticoagulants taken in patients with and without cerebral hemorrhage after TBI (ASA, acetylsalicylic acid; LMWH, low-molecular-weight heparin; NOAC, novel oral anticoagulants; PAI, platelet aggregation inhibitor). *p ≤ 0.05, **p ≤ 0.01, and ***p < 0.001.

Patient charts were screened for documented premedication, especially regarding the type of anticoagulant (Figure 1). ASA was the most prescribed anticoagulant in these geriatric patients with TBI. ASA was found without significant difference (*p* = 0.539) in the premedication of 70.6% (*n* = 12) patients with and 77.1% (*n* = 162) patients without bleeding. A total of 34 trauma patients were on new oral anticoagulants (NOAC), of whom 8.8% (*n* = 3) presented with intracerebral hemorrhage and 91.2% (*n* = 31) did not (*p* = 0.748). Five patients took coumarins (Vitamin K antagonists, phenprocoumon/warfarin). Another 29 of the 227 patients were on other platelet aggregation inhibitor than ASA [(PAI), like clopidogrel or ticagrelor] but none of them showed higher risk for post-traumatic hemorrhage (*p* > 0.1). In total, 9 of 227 (4%) patients were taking low-molecular-weight heparin (LMWH). Among them intracerebral hemorrhage was significantly more frequent (23.5 vs. 2.4%, *p* < 0.001).

### Subdural hematoma is the most common bleeding entity in geriatric patients

**FIGURE 2:**
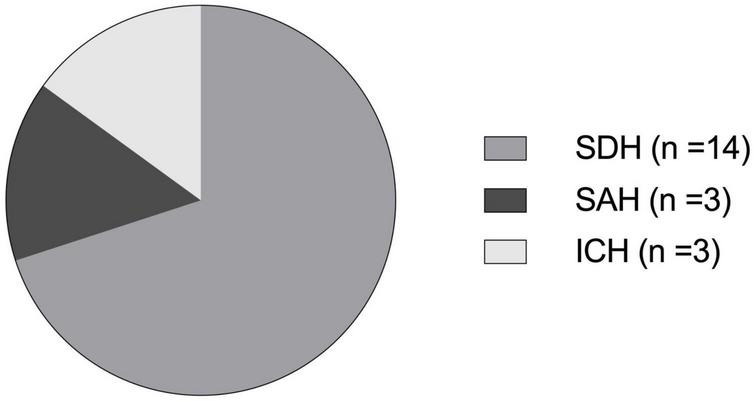
Distribution of different bleeding entities of geriatric patients (≥ 65 years) after TBI. In some patients, different bleeding entities were present simultaneously: SDH, subdural hematoma (*n* = 14); SAH, subarachnoid hemorrhage (*n* = 3); ICH, intracerebral hemorrhage (*n* = 3).

**FIGURE 3:**
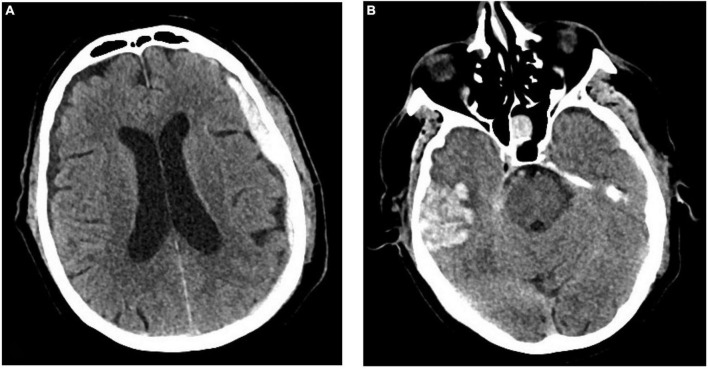
Exemplary cranial CT images of a 73-year-old male patient admitted to the emergency department of the University Hospital Frankfurt after a ground-level fall under ASA premedication. The CT scan shows the simultaneous presence of a subdural hematoma **(A)** and an intracerebral hemorrhage **(B)**. ©Department of Diagnostic and Interventional Radiology, University Hospital Frankfurt, Frankfurt am Main, Germany.

Figure 2 shows the distribution of different bleeding entities of geriatric patients (≥ 65 years) after TBI. In total, 17 of 227 patients suffered intracerebral hemorrhage after TBI. In 12 patients a subdural hematoma (SDH, Figure 3) was documented, 2 patients suffered subarachnoid hemorrhage (SAH), and 3 scans showed an intracerebral hemorrhage (ICH, Figure 3) with simultaneous occurrence of SDH (*n* = 2) and SAH (*n* = 1), respectively.

### Amnesia is the most sensitive major symptom for intracerebral hemorrhage after TBI in elderly

**FIGURE 4:**
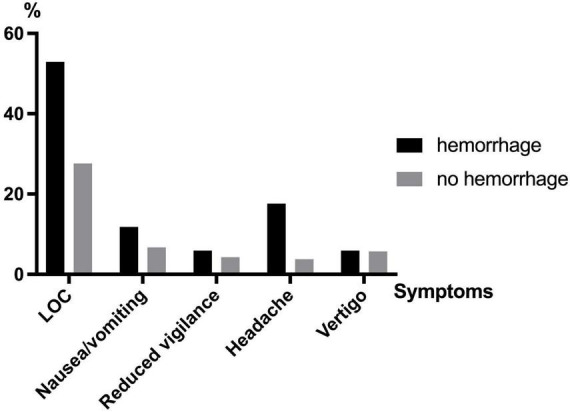
Major and minor symptoms in patients with and without cerebral hemorrhage after TBI (LOC, loss of consciousness).

The anamnesis and first clinical examination of the patients were analyzed for major [amnesia, loss of consciousness (LOC), vomiting] and minor (headache, dizziness) symptoms of TBI (Figure 4). In total, *n* = 67 patients suffered from at least one of the aforementioned symptoms, of which 7.5% had hemorrhage. Overall, patients with cerebral hemorrhage showed significantly more neurological symptoms than patients without hemorrhage (52.9 vs. 27.5%, *p* = 0.028). However, 8 of 17 (47.1%) patients did not show any symptoms despite the detection of cerebral hemorrhage. Patients with post-traumatic hemorrhage were significantly more likely to have amnesia (17.6 vs. 3.8%, *p* = 0.11). LOC (6.7 vs. 11.8%, *p* = 0.430) and vomiting (4.3 vs. 5.9%, *p* = 0.758) occurred with similar frequency in both cohorts. Minor symptoms such as headache (5.7 vs. 5.9%, *p* = 0.977) and dizziness (2.6 vs. 0%, *p* = 0.520) were also reported with similar frequency. Considering the different bleeding entities, patients with SDH suffered significantly more often from minor intracranial pressure symptoms (13.4% > 3.8%, *p* = 0.007). The remaining entities SAH and ICH showed no relevant difference to the clinical examination results from the patients without hemorrhage. Patients with post-traumatic symptoms after TBI were hospitalized significantly more often independent from the proof of an intracranial hemorrhage (47.8 vs. 31.3%, *p* = 0.018). In asymptomatic patients (*n* = 160) who received CT for minor trauma due to existing anticoagulation, bleeding was detected in 8 patients (5%). In this group, 107 patients were taking ASA at prophylactic doses, among whom 5 patients (4.7%) suffered bleeding. The eight patients who suffered from an intracranial bleeding underwent intensive care therapy, no further patient lacking of symptoms was treated on ICU. In this subgroup, 42 patients were treated as outpatients (26.3%).

## Discussion

This retrospective study analyzed data of 227 geriatric patients of a level-1 trauma center over a 3-year period with TBI who underwent a subsequent cCT scan because of anticoagulant premedication. In 17 patients, cCT examination showed post-traumatic intracerebral hemorrhage, of which SDH was the most common. ASA and NOAC use were not associated with increased bleeding risk. But in about half of these patients, bleeding occurred even in the absence of symptoms, which accounted for 5% of all symptom-free patients. Of 160 asymptomatic patients, 107 patients were taking ASA at prophylactic doses, of whom 5 patients (4.7%) experienced bleeding.

Life expectancy is increasing, which results in a higher number of accidents in the geriatric age group (≥ 65 years). In addition to relevant accident mechanisms such as falls from great heights or traffic accidents, accidents with minor injury mechanisms like ground level falls often result in relevant injuries in this group (16, 17). In this study men and women were almost equally affected by post-traumatic intracranial hemorrhage, and the mean age of both cohorts was 81 years of age. Based on a prior analysis of admission diagnoses from 2019 at the University hospital Frankfurt, it was shown that the elderly suffer head injuries at a 1:1 ratio between men and women (8). In most cases, a multi-functional gait disorder is present, usually caused by risk factors such as decreased strength, coordination disorders, and visual impairment. In geriatric traumatology, the focus has been on main diagnoses such as fractures of the femur, the pelvis, or the spine (18, 19). Meanwhile, TBI after a fall, is one of the most common injury entities in patients of advanced age (6).

The most important finding of this study answers the question whether routine cCT in elderly patients on permanent anticoagulants, is statistically and medically appropriate. The results with regard to ASA is particularly interesting. In addition to NOAC and the combination of PAIs, which are mostly used therapeutically, the preventive use of ASA is very common and discussed critically. ASA is an integral part of the secondary prevention of cardiovascular disease. Patients who have already suffered a myocardial infarction or ischemic stroke are usually prescribed ASA at doses of up to 100 mg/day. For primary prevention in patients, however, ASA administration is controversial. Especially in Anglo-American countries, but also in Germany, the uncritical use of ASA in the primary prevention of cardiovascular disease is widespread. Among them, almost half of those over 70 years of age take ASA daily. In 2018 alone, three studies questioning the preventive benefits of low-dose ASA were published. In addition to the ARRIVE and ASCEND trials that compared healthy subjects with at-risk groups, the ASPREE (“Aspirin in Reducing Events in the Elderly”) trial analyzed population over an age of 65. The absolute benefit of primary prevention appears to be small, but there was evidence of an increased risk of bleeding in the elderly, as well as in all other age groups. According to the meta-analysis, there is a 31% increase in intracranial hemorrhage with ASA treatment (20). In this study, it was shown that cerebral hemorrhage is not significantly increased by the use of ASA, NOAC or other PAI. Nevertheless, even in the absence of any neurological symptoms, about 5% of the patients in our study on prophylactic ASA medication showed an intracranial bleeding, which strengthens the necessity to perform cCT even following minor head trauma in elderly patients on any anticoagulant medication.

Special care is required in the diagnosis of older patients with TBI (18). Intracranial hemorrhages may remain masked for a long time, especially in elderly patients. It has been described that the use of anticoagulants is associated with an increased risk of bleeding, especially due to traumatic causes. And the use of anticoagulant medications is associated with a high risk of occult intracranial hemorrhage, i.e., without correlating symptoms (5, 10). Thus, the major challenge in diagnosing acute cerebral hemorrhage in the elderly is that the patient’s initial symptoms often do not match the radiologic findings (21). It is not surprising that cCT is also performed significantly more often after admission to the ED in those over 65 years of age who are significantly more likely to have relevant preexisting conditions and anticoagulation (22). The diagnostic and therapeutic approach to TBI is initially based on the accident mechanism, the presence and severity of neurologic symptoms, and depending on existing risk factors. Patients with moderate to severe TBI (GCS < 13 points) usually undergo immediate cCT to quickly diagnose a possible intracranial injury (10). A 2017 study recommends routine cCT after a fall, especially in all patients older than 85 years. Although all 737 study participants were clinically stable and had a GCS of 15, 437 patients underwent cCT after clinical examination, which revealed intracranial hemorrhage in one third of the patients (21). According to statistics from the Federal Office of Germany, approximately 165,600 patients > 65 years of age were hospitalized nationwide in 2018 due to a head injury sustained in any accident (23, 24). In this study patients presenting with post-traumatic symptoms after TBI were hospitalized significantly more often. Even though patients with documented hemorrhage had a significant longer in-hospital stay and ICU treatment. For elderly, rapid recovery is essential because mobility and independence are more difficult to regain than in younger patients. However, it is essential for avoiding and minimizing the need for long-term care (25). There is professional discourse but no clear guideline yet to perform cCT in patients with mild or no symptoms of TBI who are taking anticoagulant medications to rule out possible intracranial hemorrhage (10, 21, 26). Despite minor trauma mechanisms, 17 of 227 patients in this study experienced cerebral hemorrhage. However, in almost 50% of these cases, an intracranial bleeding was detected despite the presence of clinical symptoms. Among these, retrograde amnesia occurred significantly more frequent in patients with post-traumatic hemorrhage. Retrograde amnesia is a mostly temporary form of memory loss regarding events that occurred after the causative event for the amnesia. It is one of the major symptoms of acute brain injury (6). In the elderly, clinical occult hemorrhages occur more frequently because symptoms of increased intracranial pressure may develop later due to the already reduced brain mass. Especially SDH, which is the most common entity of cerebral hemorrhage in the elderly, just like in this study, may remain asymptomatic for a longer time due to its pathophysiology in reduced brain mass (12, 27). Whereby certainly a relevant proportion of geriatric already suffer relevant limitations of memory and retrograde amnesia must be discussed in this context.

Of particular interest were the results showing that the use of NOACs did not lead to increased rate of bleeding in the included patients. However, due to the small number of cases compared to ASA, no conclusive statements can be made. Although we demonstrated an increased incidence of intracranial hemorrhage with LMWH therapy in this study, outpatient use of this agent is uncommon and, to that extent, has reduced validity for the general use of a cCT in geriatric TBI patients on anticoagulation.

On the basis of the data presented here, we continue to believe that the calculated use of cranial CT in geriatric patients with TBI on anticoagulation cannot be dispensed. This is mainly due to the high number of clinically occult hemorrhages that were only diagnosed by cCT. And especially under ASA, which is currently used inflationary in cardiovascular prophylaxis, a not negligible number (5%) of intracerebral bleeding occurred.

### Limitations of the study

The most important limitation is the retrospective nature of the data analysis. Another limitation is the single center study design, which only reflects the urban demographics of a large city. This may have a limiting influence on the generalizability of our study results, and it is possible that these results are not applicable to all trauma situations. Overall, the number of positive findings was low, limiting the comparability of patients with intracranial hemorrhage. Nevertheless, the main message of this study is supported by the positive results, because in these cases there is a relevant change in the clinical procedure, such as monitoring in the hospital and the basic risk or general indication for CT in ASA and NOAC intake.

## Conclusion

Acetylsalicylic acid and NOAC use are not associated with increased bleeding risk in geriatric trauma patients (≥ 65 years) after fall-related TBI. Nevertheless, in almost 50% of cases, intracranial bleeding occurs even in the absence of neurological symptoms, independent from the type of anticoagulant medication. Therefore, cCT is a mandatory tool to exclude cerebral hemorrhage in elderly patients on anticoagulation.

## Data availability statement

The raw data supporting the conclusions of this article will be made available by the authors, without undue reservation.

## Ethics statement

This study has been conducted after approval by the Institutional Review Board of the University Hospital of the Goethe University Frankfurt (2021-90).

## Author contributions

PS designed the study, established the methods, performed the statistical analysis, and revised the manuscript. CS carried out data analyses, obtained the ethical approval for human analyses, performed the statistical analysis, and wrote the first draft of the manuscript. AB collected data and carried out data analyses. IM and KE critically reviewed the manuscript. MW and RV contributed intellectually to the completion of the study. All authors contributed to the article and approved the submitted version.
